# Rare Insights: Temporoparietal Necrotizing Fasciitis Stemming From a Dental Source

**DOI:** 10.7759/cureus.60278

**Published:** 2024-05-14

**Authors:** Chetan Gupta, Nitin Bhola, Parmarth Sonpal, Nidhi Bodiwala

**Affiliations:** 1 Oral & Maxillofacial Surgery, Sharad Pawar Dental College & Hospital, Wardha, Wardha, IND

**Keywords:** odontogenic, anesthesia, infratemporal, fascial spaces, necrotizing fasciitis (nf)

## Abstract

Necrotizing fasciitis (NF) of the face is a rare yet serious condition requiring prompt and comprehensive management. This approach typically involves input from various medical specialties such as infectious disease specialists, critical care physicians, and surgeons. The primary goals are early recognition, aggressive surgical debridement, appropriate antibiotic therapy, and supportive care. Prompt diagnosis is crucial, based on symptoms like severe pain, rapidly spreading erythema, and systemic signs of infection. Broad-spectrum antibiotics are initiated empirically, and adjusted based on culture results. Urgent surgical debridement is crucial, removing all necrotic tissue. Careful consideration must be given to preserve vital structures. Close monitoring and intensive care may be necessary, especially for severe cases. Soft tissue reconstruction may follow once the infection is controlled, aiming to restore function and aesthetics. Long-term follow-up is essential to observe for complications and recurrence.

## Introduction

Necrotizing fasciitis (NF) is a severe soft tissue infection primarily spreading through fascial planes. It is usually accompanied by systemic inflammatory response syndrome (SIRS) and needs prolonged intensive care treatment [1]. Although necrotizing fasciitis can affect various parts of the body, including the extremities, abdomen, and perineum, it can also arise in the oro-pharyngeal and maxillofacial regions [2]. In the head and neck region, odontogenic causes are most prevalent, leading to rapid tissue infiltration, vascular compromise, and potential organ failure or limb loss [3]. The condition is commonly referred to as the "flesh-eating disease" due to its aggressive nature and ability to cause extensive tissue destruction. Early NF diagnosis is challenging due to subtle symptoms unless triggered by toxic shock syndrome or organ failure [3]. Advanced cases manifest with significant tissue loss, vascular complications, or systemic involvement, often leading to fatal outcomes. If not treated at once with appropriate drugs or surgery, it may spread through the bone marrow and cortex, reaching muscle layers, fascial spaces, and thence various vital organs [4]. Aggressive forms are often the result of underestimation of the infection, late diagnosis, erroneous surgical approach, and septic complications. However, NF involving the temporalis muscle remains infrequently reported. In the current study, we present a case of Temporal Necrotizing Fasciitis due to a suspected odontogenic cause in a geriatric patient.

## Case presentation

**Figure 1:**
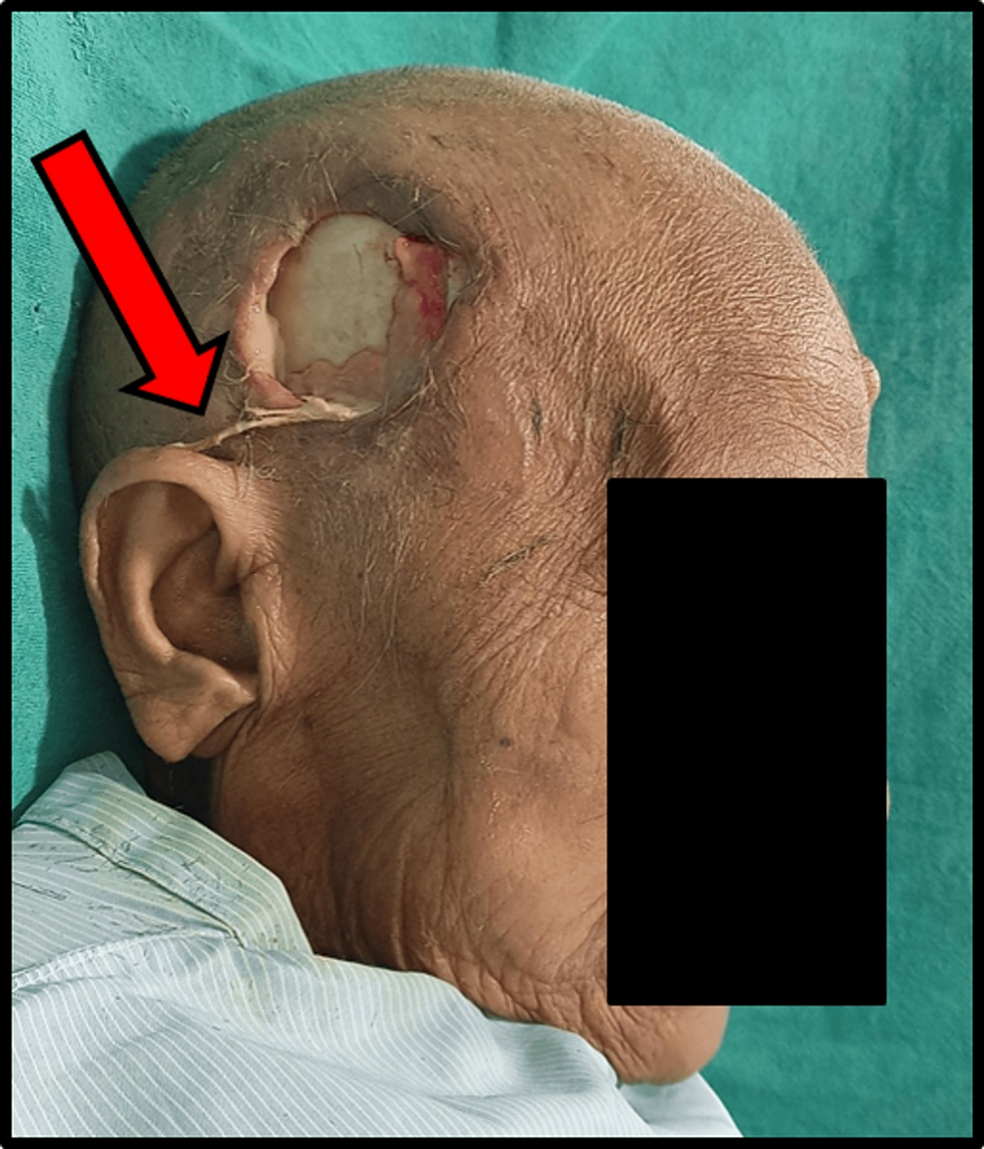
Pre-operative image showing necrotizing fasciitis over right temporal region Pre-operative image showing necrotizing fasciitis over right temporal region with sloughed margins and exposed temporal bone.

A 77-year-old female patient presented to our outpatient department with the complaint of severe pain and pus discharge from the right side of the head (Figure 1) with general malaise in the past 10-15 days approximately. The pain was gradual in onset, throbbing, continuous, and localized in nature. While inspecting inflamed, slough tissue with draining pus from the right tempo-parietal and right retromolar region.

**Figure 2:**
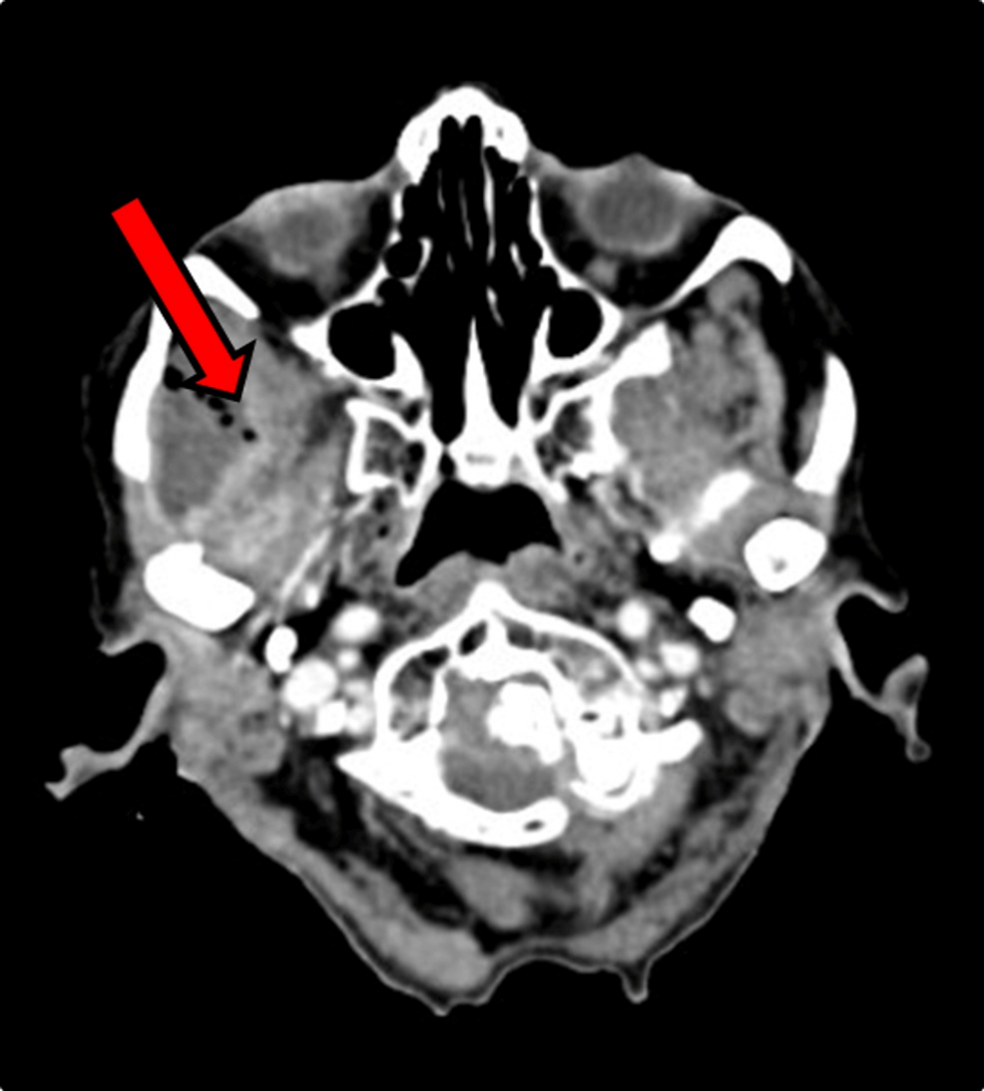
Axial Section of Contrast-Enhanced Computed Tomograph (CECT) Head There is evidence of peripherally enhancing collection with internal enhancing septations within noted over the right frontoparieto temporal regions with multiple air density foci within. The collection approximately measures 9.5 x 2.9 x 12 cm (Axial Plane Topography with Volume Computerized Tomography Contrast (APTVCC)). Inferiorly collection is seen extending into the right infratemporal fossa, masticator, and submandibular spaces. There is involvement of the medial pterygoid and superficial and deep fibers of the temporalis muscle. Masseter muscle also shows heterogeneous enhancement and is mildly bulky as compared to the opposite side.

**Figure 3:**
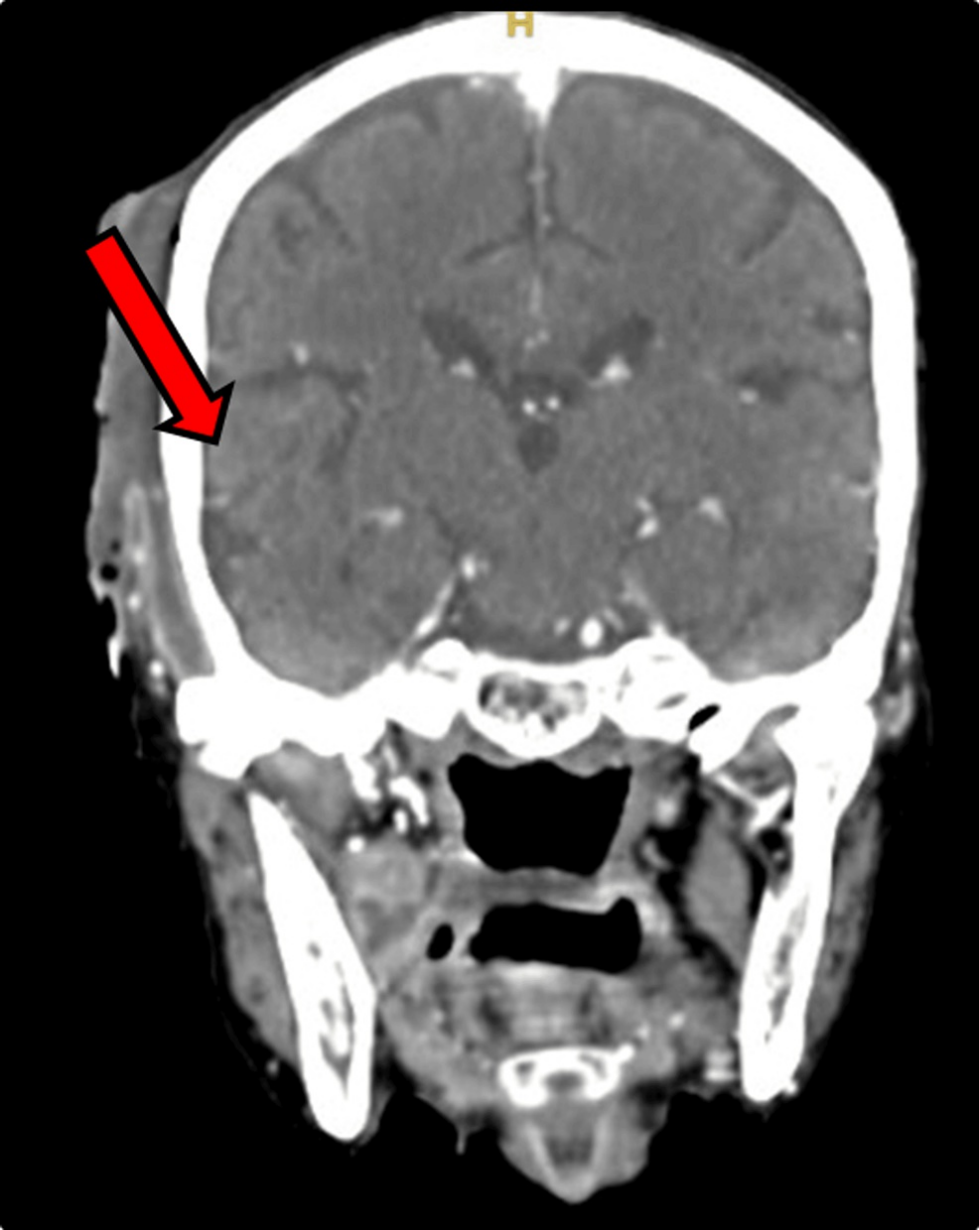
Coronal Section of Contrast-Enhanced Computed Tomograph (CECT) Head There is evidence of peripherally enhancing collection with internal enhancing septations within noted over the right frontoparieto temporal regions with multiple air density foci within (coronal section).

Upon examination, there was crepitus and fluctuant swelling noted in the right temporo-parietal region. A thorough intra-oral examination was done for the patient upon which a grossly decayed tooth in the lower right mandibular region was found. Contrast Enhanced Computed Tomograph (CECT) of the Head (Figure 2, Figure 3) was done for this patient, which showed evidence of peripherally enhancing collection with internal enhancing septations noted over the right fronto-parieto-temporal regions with multiple air density foci within. The collection approximately measured 9.5 x 2.9 x 12 cm. Inferiorly collection is seen extending into the right infratemporal fossa, masticator, and submandibular spaces.

The general condition of the patient was poor with severe nutritional cachexia, Body Mass Index of 10.82, reduced air entry in the bilateral lower lobe, and crepitus in the left lower lobe of the lung. Following routine investigations were sent for the patient (Table 1).

**Table 1:** Pre-operative Investigations NGSP: National Glycohemoglobin Standardization Program; HbA1c/A1C: glycated hemoglobin; ADA: American Diabetes Association

Investigation	Observed Value	Biological Reference Range	Method
Haemoglobin (Hb)	6.8 gm%	Male: 13-17 gm% Female: 12-15 gm%	Photometric Measurement
Total WBC Count	23500/cu.mm	Male: 4000-10000 cu.mm Female: 4000-10000 cu.mm	Coulter Principal
Red Blood Cells (RBCs)	2.79 millions/cu.mm	Male: 4.5-5.5 millions/cu.mm Female: 3.8-4.8 millions/cu.mm	Coulter Principal
Total Platelet Count	6.91 Lacs/cu.mm	Male: 1.50-4.10 Lacs/cu.mm Female: 1.50-4.10 Lacs/cu.mm	Coulter Principal
HbA1c	4.0 % A1C NGSP	Non Diabetic- <6.0 % A1C NGSP, Action suggested- >7.0 % A1C NGSP, ADA Target- 6.0-7.0 % A1C NGSP	Enzymatic

The patient was planned for surgical drainage of space infection under general anesthesia. After pre-anesthetic checkup high risk and poor prognosis fitness was obtained. The patient’s relatives were not willing to get operated on under general anesthesia, hence it was planned under local anesthesia. Before starting the antibiotics, we sent pus from the extraoral temporoparietal and intraoral retromandibular region for the culture and sensitivity test. We initiated empirical antibiotic therapy for the patient until the final culture report was available.

**Figure 4:**
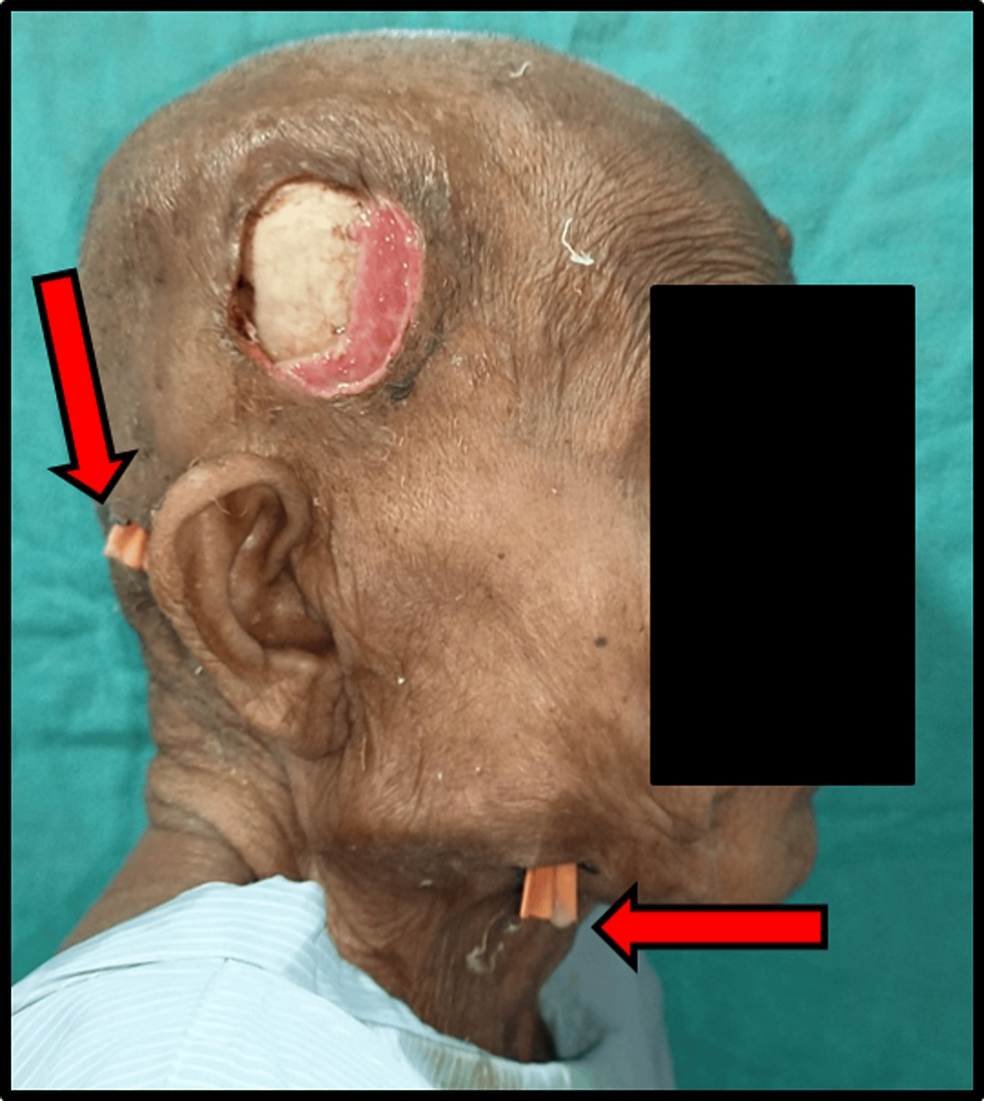
Post-operative image showing corrugated rubber drain placement over the right temporal and submandibular region It is also showing signs of healing over the right temporal region.

Incision and drainage under local anesthesia were done. Right auriculotemporal nerves, zygomaticotemporal, lesser occipital, posterior auricular nerve and mandibular nerve blocks were given. Incision and drainage of the right superficial temporal, deep temporal, buccal, lateral pharyngeal, and submandibular space were done. The extraction of 2nd and 3rd right mandibular molar teeth with through curettage was done. The dead and decayed soft tissue debridement is done meticulously. The submandibular space was explored and through the same incision lateral pharyngeal space exploration and drainage followed by corrugated rubber drains (Figure 4) were placed in respective areas.

The patient started on IV antibiotics according to the blood counts. After 24 hours of incubation, no growth was there in the sent specimen for culture sensitivity. Post-operative blood transfusion and supportive care were given. There was an improvement in the patient’s general condition the post-operative routine labs are given in Table 2.

**Table 2:** Postoperative Investigations

Investigation	Observed Value	Biological Reference Range	Method
Haemoglobin (Hb)	9.2 gm%	Male: 13-17 gm% Female: 12-15 gm%	Photometric Measurement
Total WBC Count	11500/cu.mm	Male: 4000-10000 cu.mm Female: 4000-10000 cu.mm	Coulter Principal
Red Blood Cells (RBCs)	3.55 millions/cu.mm	Male: 4.5-5.5 millions/cu.mm Female: 3.8-4.8 millions/cu.mm	Coulter Principal
Total Platelet Count	6.29 Lacs/cu.mm	Male: 1.50-4.10 Lacs/cu.mm Female: 1.50-4.10 Lacs/cu.mm	Coulter Principal

Due to some circumstances, the patient’s relatives discontinued the treatment and took discharge against medical advice.

## Discussion

The infection of the second or third mandibular molar is the frequent reason for odontogenic necrotizing fasciitis (NF) [5]. This infection may travel cranially up to the skull base or caudally towards the thorax [6]. The mortality rate for NF patients is 9.8%, without diabetes mellitus [7]. NF can result from various bacteria, including *Streptococcal*, *Staphylococcal*, *Fusobacterium*, or *Acinetobacter *species, with polymicrobial infections being common [8]. Bacterial infiltration causes tissue necrosis and gas formation in subcutaneous, fascial, and deep tissues [9]. In the present case the infection spread from an abscess in the right submandibular region to the peripharyngeal fascia and the external pterygoid muscle, and along the ramus to the right zygomatic area, involving the soft tissue of the temporoparietal region In this case, thorough drainage of affected spaces was performed to prevent further tissue destruction and systemic complications. Additionally, the placement of corrugated rubber drains aids in continuous drainage and prevents re-accumulation of pus. Prompt and aggressive surgical debridement is essential to remove necrotic tissue and control the spread of infection. The use of broad-spectrum intravenous antibiotics is crucial to target the causative bacterial pathogens effectively [10]. Considering the poly-microbial nature of NF, a combination of antibiotics targeting both aerobic and anaerobic bacteria is often recommended. The initiation of antibiotics alongside surgical intervention helps to eradicate bacteria systemically and reduce the risk of systemic complications [11]. Early recognition and aggressive management are essential to mitigate adverse outcomes. Treatment involves aggressive surgical debridement alongside IV antibiotics, with some patients receiving hyperbaric oxygen therapy. Prompt recognition, extensive debridement, and broad-spectrum antibiotics are crucial for preventing severe morbidity and mortality [12].

## Conclusions

Necrotizing fasciitis originating from odontogenic sources poses a grave threat, necessitating prompt intervention to avert severe complications. Given the significant mortality and morbidity associated with NFs, it's pertinent to consider the saying, "Surgery is often the most effective antibiotic". Further research is imperative to enhance diagnostic and therapeutic strategies for improved patient outcomes.
